# Green Anisole as Antisolvent in Planar Triple-Cation Perovskite Solar Cells with Varying Cesium Concentrations

**DOI:** 10.3390/mi15010136

**Published:** 2024-01-15

**Authors:** Vera La Ferrara, Antonella De Maria, Gabriella Rametta

**Affiliations:** Italian National Agency for New Technologies, Energy and Sustainable Economic Development (ENEA), Portici Research Center, 80055 Portici, Italy; antonella.demaria@enea.it (A.D.M.); gabriella.rametta@enea.it (G.R.)

**Keywords:** green antisolvent, perovskite, triple cation, anisole, stability, planar solar cell, unencapsulated, cesium, ambient air

## Abstract

The feasibility of replacing toxic chlorobenzene antisolvents with environmentally friendly anisole in the fabrication of planar triple-cation perovskite solar cells was explored here. The successful integration of anisole not only ensures comparable device performance but also contributes to the development of more sustainable and green fabrication processes for next-generation photovoltaic technologies. Nevertheless, to ensure the possibility of achieving well-functioning unencapsulated devices whose working operation depends on outdoor atmospheric conditions, we found that adjusting the cesium concentrations in the perovskite layers enabled the electrical characterization of efficient devices even under high relative humidity conditions (more than 40%). We found that 10% of CsI in the precursor solution will make devices with low hysteresis indexes and sustained performance stability over a 90-day period both with cholorobenzene and anisole antisolvent. These results further confirm that green anisole can replace chlorobenzene as an antisolvent.

## 1. Introduction

The swift advancement witnessed in perovskite solar cells (PSCs) over the last decade has positioned them as the most promising next-generation photovoltaic technology. Remarkable strides in efficiency and stability have been showcased in laboratory settings, yet substantial efforts are imperative to facilitate the seamless transition of printable PSC technology from the lab to industrial-scale applications for commercialization. Among the various objectives required to achieve complete commercialization, the use of eco-friendly solvents is a priority in creating devices whose properties are increasingly less affected by the surrounding environment, ensuring greater stability of the initial efficiency. In the design process of standard PSCs, a device stack, composed of at least three layers, is crafted using solution-based techniques. Consequently, the careful selection of appropriate solvents for depositing each functional layer takes on paramount significance. Solvent systems, which are indispensable for each functional layer, must meet and adhere to two pivotal criteria: firstly, they should be non-hazardous in order to align with the rigorous standards of industrial manufacturing, and secondly, they should not compromise the structural integrity of the underlying layers. The deposition of perovskite absorber layers (PALs) with antisolvent engineering is a highly common method employed in perovskite photovoltaics research [1]. The assisted spin-coating method is the most common strategy to obtain high-quality perovskite film. Here, the spin-coating of the perovskite precursor solution employed an antisolvent treatment to facilitate the removal of the host solvents and initiate the crystallization of the perovskite film. Nevertheless, the use of antisolvents can raise problematic issues, including chemical hazards and poor compatibility. In terms of hazardous waste, the amount of antisolvent released into the ambient environment is typically four to five times as high as that of the solvent used to prepare the perovskite. If the mass production of PSCs reaches the stage where gigawatts of energy are produced, the tons of antisolvents released into the air during their fabrication will raise serious safety concerns [2]. It is noteworthy that the majority of high-efficiency PSCs in operation employ chlorobenzene (CB) as an antisolvent. Chlorobenzene contains a benzene ring structure, which classifies it as an aromatic compound, and it also contains chlorine atoms bonded to the benzene ring, making it a halogenated compound. Indeed, chlorobenzene can have negative environmental and health impacts. It is essential to underscore that the widespread use of highly toxic halogenated solvents, such as CB, is suboptimal for large-scale industrial production, which is primarily due to their adverse impacts on both human health and the environment.

Lately, various eco-friendly antisolvents have emerged as potential alternatives to hazardous chlorobenzene [3,4]. Butanol, ethyl acetate and anisole are some of the green antisolvents considered for the fabrication of perovskite solar cells in various research studies and applications [5,6,7]. These solvents were chosen for their relatively lower toxicity, reduced environmental impact and compatibility with perovskite materials, making them more environmentally friendly alternatives to traditional solvents like chlorobenzene. When considering the replacement of CB, it is crucial to evaluate the properties of antisolvents, including factors like, for example, the boiling point, relative polarity and solubility in water. In Table 1, the main properties of these antisolvents are reported together with the ICH class (International Council for Harmonisation of Technical Requirements for Pharmaceuticals for Human Use) that indicates the recommended limits in the use of solvents: Class 1 solvents are to be avoided; Class 2 solvents are to be limited and Class 3 solvents have low toxic potential. Chlorobenzene belongs to Class 2, while butanol, anisole and ethyl acetate belong to Class 3; therefore, no health-based exposure limit is needed.

Despite high-efficiency perovskite-based solar cells having been achieved using butanol and ethyl acetate as antisolvents [5,6], both pose limitations for large-scale production.

The high relative polarity and water solubility of butanol may negatively impact device stability when exposed to higher humidity in ambient air [2]. Additionally, ethyl acetate is hindered by a low boiling point, rendering it unsuitable for scalable production. This is because the low boiling point and subsequent high volatility will not only result in undesirable air emissions but also present a significant risk of worker exposure to hazardous substances. Manufacturing processes that utilize antisolvents with low boiling points require stringent control measures to prevent the formation of explosive vapor mixtures. Consequently, extremely volatile antisolvents are not the preferred choice in such processes.

Nevertheless, anisole (ANI) is a potential green antisolvent that can replace chlorobenzene, since their properties are very similar (Table 1). Anisole is an aromatic compound that, when used as antisolvent during perovskite deposition, can protect the intermediate and nucleation phases from the irregular growth of the perovskite film, guaranteeing its uniformity. Presumably, ANI quickly extracts the solvents of the precursor solution, typically DMF and DMSO, from perovskite surface during the dripping [8]. Despite anisole being already used instead of CB in the years 2017–2018 [8,9,10,11,12], it is worth noting that the importance of replacing chlorobenzene with anisole has not been fully recognized yet, because in today’s scientific papers, CB continues to be used and only a few works use anisole as antisolvent in the n-i-p architecture [2,8,9,10,11,12]. Interestingly, anisole has been used as solvent in hole transport material (HTM) [13]. In Table 2, scientific papers are listed where anisole was used as an antisolvent for n-i-p triple cation perovskite deposited onto an electron transport material (ETM) such as compact or mesoporous TiO_2_ or for double cation on tin dioxide. To the best of our knowledge, there are no scientific studies on the use of anisole as an antisolvent for a triple-cation perovskite deposited on planar substrates with tin dioxide as an ETM.

In this study, we strongly emphasize the possibility of substituting toxic CB with green anisole by realizing efficient planar triple-cation perovskite solar cells on SnO_2_ as an ETM. In addition, to ensure the feasibility of achieving efficient unencapsulated devices, whose operational performance relies on outdoor atmospheric conditions, we discovered that adjusting cesium concentrations in perovskite layers allowed for the electrical characterization of well-functioning devices even under high relative humidity conditions (exceeding 40%), throughout the year, with changes in temperature and relative humidity. It is well known that the cesium concentration makes the triple-cation perovskite composition thermally more stable as long as it has fewer phase impurities and is less sensitive to processing conditions [14,15]. Indeed, higher Cs concentrations resulted in well-functioning PSCs, which are less affected by fluctuating surrounding variables such as temperature and relative humidity (RH); in our laboratory during the measurement period, spring to summer time, they were in the range of 10–37 °C and 45–93%, respectively. A low hysteresis index and good performance stability over the 90 days were observed for devices with higher Cs concentrations both with CB and ANI antisolvents. However, when anisole was used as an antisolvent, the devices exhibited even lower hysteresis, maintaining 80% of their overall power conversion efficiency (PCE). This confirms that environmentally friendly anisole can replace toxic CB.

## 2. Materials and Methods

### 2.1. Materials

SnO_2_ colloid precursor (tin(IV) oxide 15% H_2_O colloidal dispersion), PbI_2_ (≥99.999, ultradry) and PbBr_2_ (Puratronic, ≥99.998) were obtained from Alfa Aesar. CsI (≥99.999, anhydrous) and acetonitrile (ACN) were obtained from Acros Organics. Anisole (≥99.7 anhydrous) was purchased from Sigma Aldrich. N,N-dymethylformamide (DMF) (Merk life science s.r.l., Milano, Italy), dimethyl sulfoxide (DMSO), formamidinium iodide (FAI), methylammonium bromide (MaBr, ≥99%, anhydrous), Spiro-OMeTAD, chlorobenzene (CB), 4-tert-butylpyridine (TBPy), bis(trifluoromethane)sulfonimide lithium salt (Li-TFSI), and FK 209 Co(III) TFSI salt were purchased from Merck. All the chemicals were used without further purification. We used 2 × 2 cm^2^ glass/ITO substrates received from Kintec (10 Ω sq^−1^).

### 2.2. Solutions

The SnO_2_ solution was prepared by diluting the commercial SnO_2_ colloidal dispersion with deionized water (1:5). The perovskite Cs_x_(MA_0.17_FA_0.83_)_(1−x)_Pb(I_0.83_Br_0.17_)_3_ (mixed cation Cs, FA = formamidinium, MA = methylammonium, and mixed halide (I,Br)) solution was prepared by mixing PbI_2_ (1.10 M), PbBr_2_ (0.22 M), FAI (1.05 M), and MABr (0.20 M) in DMF/DMSO (4 : 1 *v*/*v*%), and the CsI iodide solution (1.50 M) in DMSO was added to the above solution to obtain a molar ratio of 5% and 10%.

The hole transport material (HTM) solution consists of 73 mg of Spiro-OMeTAD diluted in 1 mL of CB, 27 µL of TBPy, 17 µL of a Li-TFSI solution (520 mg of Li-TFSI in 1 mL of ACN), and 7 µL of a FK209 Co(III) TFSI (375 mg of Co-complex in 1 mL of ACN).

### 2.3. Device Fabrication

**Figure 1:**
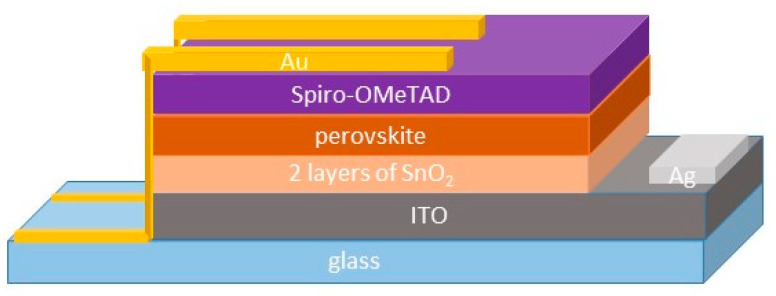
Architecture of typical device.

The ITO-coated substrates were cleaned by sequential sonication in acetone and ethanol for 15 min in each solvent and then dried using nitrogen flux. After 20 min of UV ozone treatment for ITO, two layers of SnO_2_, as electron transport materials (ETLs), were deposited using a spin coater at 6000 rpm for 30 s, under a fume extractor system, where temperature and relative humidity were monitored but not controlled, depending on the outdoor atmospheric conditions. Tin dioxide layers were annealed at 130 °C for 1 h on a hotplate and controlled using a thermocouple. The devices were treated again 30 min under UV ozone treatment and then inserted into a nitrogen-filled glovebox (O_2_ ≤ 1 ppm and RH ≤ 1 ppm) for perovskite and hole transport material (HTM) spinning deposition and the subsequent thermal evaporation of gold electrical contacts. The perovskite spin-coating process was set as a two-step program with 1000 and 6000 rpm for 10 and 20 s. A few seconds before spin coating was completed, 200 µL of CB or anisole, as an antisolvent, was added dropwise to the substrates. PAL was crystallized by annealing at 100 °C for 1 h. HTM solution was spun onto PAL at 4000 rpm for 30 s. Finally, the devices were completed in a thermal evaporator inside the glovebox and an 80 nm Au back-contact layer was evaporated on the HTM, using a mask to define the positive electrode. Different batches were prepared by varying the amount of CsI (5% and 10%) in the precursor solution and comparing the CB and ANI antisolvents. In the following section, we refer to the devices as Cs5 and Cs10, adding CB or ANI to distinguish the antisolvents. The schematic of the device is shown in Figure 1.

### 2.4. Device Characterization

Scanning electron microscopy (SEM) analysis was performed using a Thermo Fisher Scientific Phenom pro X SEM, with an electron beam accelerated at 15 kV, equipped with a long lifetime thermionic source of cerium hexaboride. The cross-sections used to evaluate the different layers in PSCs were made using an FEI Dual Beam Quanta 200 3D apparatus, which integrates a finely focused gallium ion beam (FIB). Transmittance spectra were measured with a Perkin Elmer λ-900 spectrophotometer. The thicknesses of the different layers were measured using a KLA Tencor profilometer. The J-V curves of the solar cells were acquired by a solar simulator, at AM1.5G, applying a shadow mask aligned to the cell area (~0.1 cm^2^). Reverse and forward scans were recorded at a rate of 2 V/s. The current–voltage characteristics of the devices were obtained by applying an external potential bias to the cell and recording the generated photocurrent using a Keithley (Model 2651A) high-power system source meter. All the samples were not polarized, and measurements were always from 1.2 to 0.1 V (reverse and forward scan). A nitrogen flow was then applied. The solar simulator is a class A single-source (ozone-free Xenon lamp) Spectrosun X25 Mark II by Spectrolab, and it is located in ambient air without controlling, but only monitoring, temperature and relative humidity. The intensity of the simulators was calibrated using a certified IEC 60904-9 compliant monocrystalline silicon solar cell with or without an infrared cut-off filter.

## 3. Results and Discussion

### 3.1. Perovskite Bandgap and Urbach Energy

**Figure 2:**
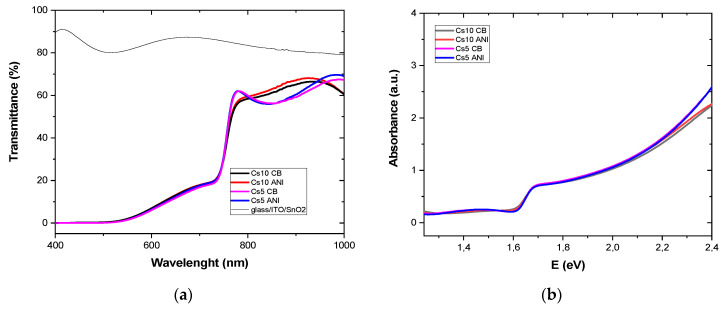
(**a**) Transmittance and (**b**) absorbance of glass/ITO/SnO_2_ w/o PAL at different antisolvents and cesium concentration.

**Figure 3:**
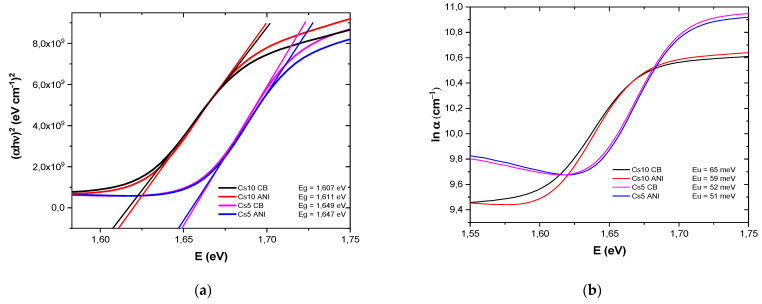
(**a**) Tauc plot and (**b**) urban energy of glass/ITO/SnO_2_/perovskite at different antisolvents and cesium concentration.

The absorption of light by semiconductors near and below their energy gap, E_g_, yields insights into the density of states and subgap states, including traps, tail states, and intermolecular species. The measurement and comprehension of subgap absorption features, particularly the Urbach energies [16], commonly utilized as an indicator of the degree of energetic disorder, offer crucial insights into the electrical and optical properties of materials, because electronic properties, including charge carrier mobility and lifetime in semiconductors, are linked to the exponential tail states. Before calculating the disorder-induced tail state absorption, it was necessary to calculate the energy gap of perovskite. It was estimated by UV-Vis absorption spectroscopy in addition to its determination from the Tauc plot. The Tauc plot is performed according to the following expression [17]:

(αhν)^m^ ∝ (hν − E_g_) (1)

where α is the absorption coefficient of the material, the quantity hν is the energy of light, and m is 2 for a direct bandgap semiconductor. Transmittance was recorded on sample glass/ITO/SnO_2_/perovskite. Transmittance (T) and absorbance (A), where A = 2−log(T), sketched in Figure 2a,b, respectively, are slightly different and dependent on cesium concentration. It can be observed that Cs5 devices exhibited higher absorption in the range of 500 and 700 nm. These data were used for the Tauc plot in Figure 3a, where α is 2303 A/d, d is the thickness of the perovskite layer, and the energy gap is the x-intercept, as reported in Table 3.

Thus, the analysis of the optical absorption in the spectral range, corresponding to the tails of the density of states, allows the determination of the Urbach energy and thereby characterizes the degree of material imperfection. The Urbach energy can be derived using the relation α = α_0_ exp(E/E_u_), where α is the absorption coefficient, E(=hν) is the photon energy, and E_u_ is the Urbach energy. The Urbach energy was calculated by plotting ln α vs. E. The reciprocal of the slopes of the linear portion, close to energy gap, gives the value of E_u_ for all the samples (Figure 3b).

Table 3 contains the bandgap and Urbach energies, E_g_ and E_u_. The energy gap depended on cesium concentration: more cesium means a smaller bandgap. Additionally, devices with more cesium had a smaller gap but exhibited a higher defect density as it was evident from larger Urbach energy. Moreover, when anisole was used as an antisolvent, E_u_ decreased for both Cs10 and Cs5, indicating a less disordered PAL and fixed cesium concentration with fewer defects.

### 3.2. SEM and FIB Analyses

**Figure 4:**
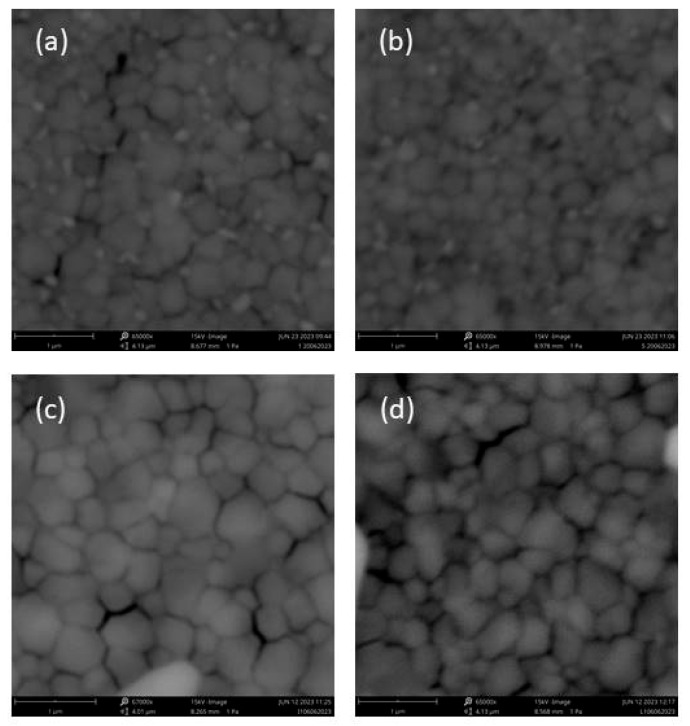
SEM images of perovskite (**a**) Cs5 CB; (**b**) Cs5 ANI (**c**) Cs10 CB (**d**) Cs10 ANI.

Figure 4 shows the surface morphologies of the PSCs with different cesium compositions and antisolvents. It is clear that the grain size depends on the concentration of Cs: as the concentration increases, the size of the grains also increases (Figure 4a,c). Moreover, there are bright small grains with 5% of Cs, whereas at 10% Cs, bright grains are not visible. In the literature, these grains are attributed to an individual PbI_2_ phase [18,19,20], which would lead to lower stability. Grain sizes were estimated using “Image Jversion 1.8.0”, which is open source software for processing and analyzing scientific images, with 324 ± 76 nm and 395 ± 85 nm for Cs10 ANI and Cs10 CB, respectively. The grain size decreased when the cesium concentration decreased, 199 ± 81 nm and 240 ± 92 nm for Cs5 ANI and Cs5 CB, respectively. Additionally, there is only a slight morphology dependence if CB or ANI are used for the same cesium concentration: ANI produces a slightly smaller grain size (Figure 4b,d).

**Figure 5:**
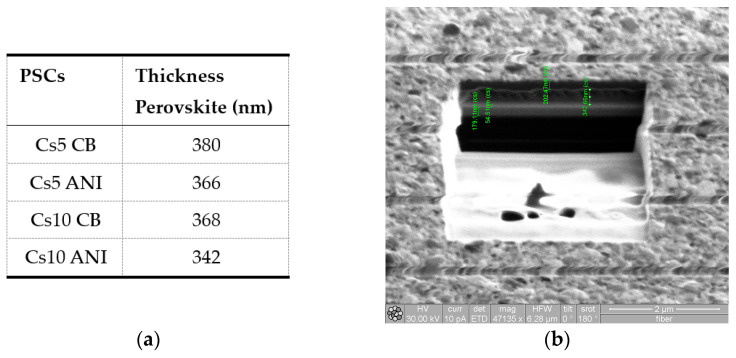
(**a**) Thickness of PALs acquired by FIB; (**b**) typical FIB cross-section of Cs10 ANI with different layers which compose the PSCs: ITO, about 180 nm, SnO_2_, about 54 nm, Cs10 ANI perovskite layer, about 342 nm and Spiro, about 200 nm.

Figure 5a contains a table with perovskite thicknesses of different devices and Figure 5b shows an ionic image of a typical cross-section, made by FIB, of Cs10. The FIB cross-section shows the instrument’s capability to section devices and assesses the thicknesses of different layers, which have also been validated using a mechanical profilometer. Different layers are evident in the image: ITO, ~180 nm, SnO_2_, ~54 nm, Cs10 ANI perovskite layer, ~342 nm and Spiro, ~200 nm.

### 3.3. J-V Characterization

To ensure the possibility of achieving well-functioning unencapsulated devices, whose working operation depends on outdoor atmospheric conditions, we found that adjusting cesium concentrations in perovskite layers enabled the electrical characterization of efficient devices even under high relative humidity conditions (more than 40%). It is well known that adding cesium as a third cation makes the triple cation perovskite composition thermally more stable as long as it has fewer phase impurities, is less sensitive to processing conditions [14,15] and is less affected by fluctuating surrounding variables such as temperature, humidity, solvent vapors or heating protocols. In fact, we found that in the presence of humidity, a higher cesium concentration indicates higher efficiency. Our unencapsulated perovskite-based devices, entirely fabricated in a glove box (O_2_ ≤ 1ppm and RH ≤ 1ppm), once the processing was finished, were characterized in an uncontrolled environment, where the J-V apparatus was located and where temperature and relative humidity strongly depended on external atmospheric conditions. Table 4 shows the temperature and RH range, corresponding to the months from April to September. These parameters show our typical working condition, and electrical measurements were acquired. During data collection, we observed that Cs5 devices, electrically characterized under RH > 40%, exhibited lower performance, despite similar devices characterized in air ambient with RH less than 30%, as reported in a previous work [21]. We have found that at RH > 40%, it was necessary to increase the cesium concentration to obtain efficient devices. In particular, Cs10 devices with anisole as an antisolvent were more efficient, which was probably because of the improved crystallization of the perovskite, making the surface less sensitive to moisture (Table 5). A similar dependence of relative humidity on the summer or winter months was also clearly stated by Li et al. [22].

**Figure 6:**
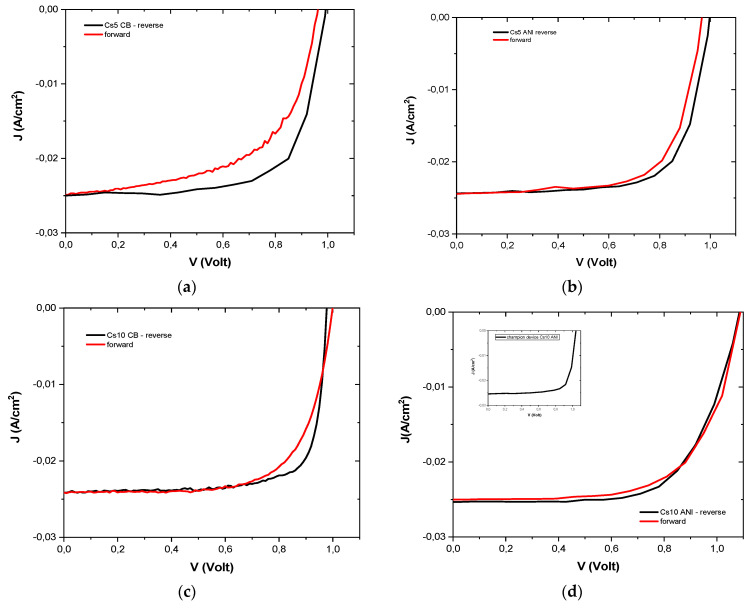
Typical J-V forward and reverse of (**a**) Cs5 CB, (**b**) Cs5 ANI, (**c**) Cs10 CB and (**d**) Cs10 ANI. In the inset, a J-V graph of champion device Cs10 ANI is shown.

The numeric value of the short-circuit current density, J_sc_, was expected to be lower for Cs5 devices when considering the value of the energy gap [23], as shown in Table 3, but it was slightly higher than that of the Cs10 device. This is probably due to the lower presence of defects, as calculated from the lower Urbach energy for the Cs5 devices (Table 3), which inhibited charge recombination. Moreover, the absorbance value (Figure 2b) was higher for the Cs5 devices, indicating the possibility of higher photon absorption and less energy loss. In Figure 6, typical J-V curves, reverse and forward, are reported. Their efficiency, in both scans, was used to calculate the hysteresis index (HI) (Equation (2) [24]), which is lower for devices with higher Cs concentrations and when ANI was used as an antisolvent, as shown in Table 6.

(2) $$Hysteresis\;index=\frac{PCE(reverse)–PCE(forward)}{PCE(reverse)}$$

**Figure 7:**
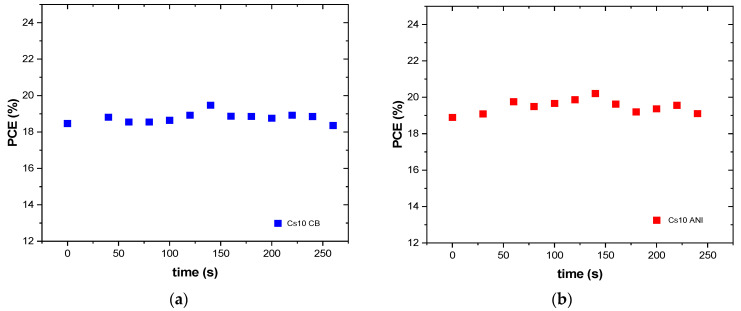
PCE measured during continuous illumination for typical devices (**a**) Cs10 CB and (**b**) Cs10 ANI.

**Figure 8:**
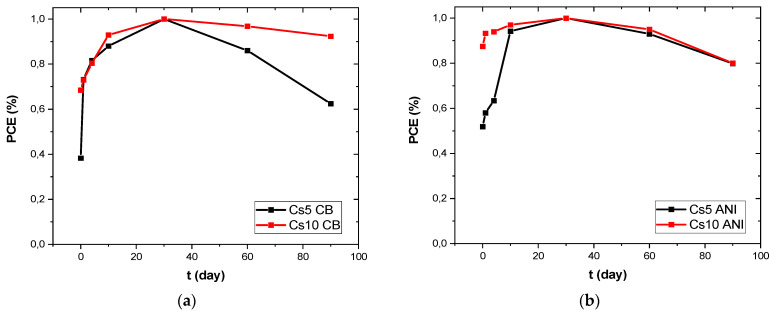
PCE monitored over 90 days: (**a**) Cs5 and Cs10 with CB as antisolvent; and (**b**) Cs5 and Cs10 with ANI as antisolvent.

We monitored these devices under continuous illumination (Figure 7) for approximately 250 s with good stability under illumination. Subsequently, the devices were monitored over 90 days (Figure 8) and stored in a low vacuum and dark chamber [21]. The initial PCE value, at zero time, was very small compared to the maximum value for both Cs5 CB and Cs5 ANI, whereas when the cesium concentration increased, the PCE was very close to the maximum value in the first measurement (red curve in Figure 8a,b). It is worth noting that the devices with anisole maintained 80% of their initial efficiency for each cesium concentration (Figure 8b).

**Figure 9:**
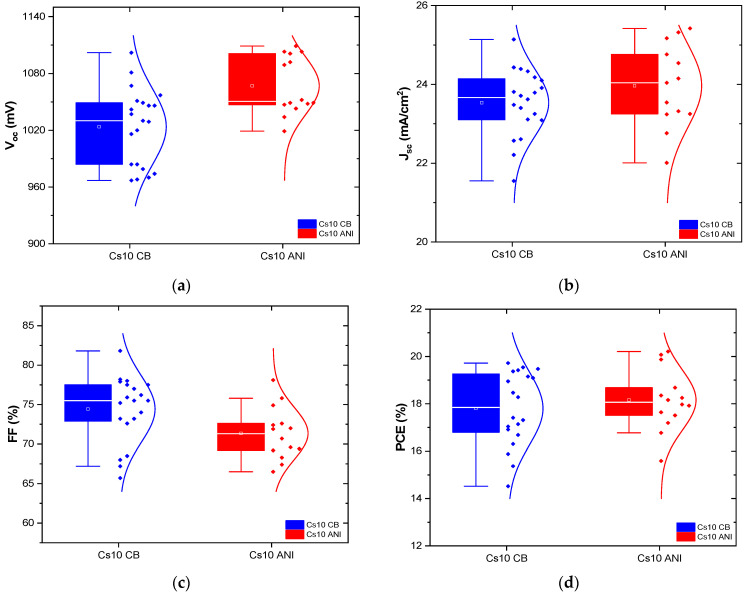
Statistics of electrical performance (**a**) open-circuit voltage, V_oc_, (**b**) short-circuit current density, J_sc_, (**c**) fill factor, FF and (**d**) power conversion efficiency, PCE, of devices Cs10 with CB (blue dots)and ANI (red dots) as antisolvents: 20 devices realized using CB and 14 devices realized using ANI. The symbol inside the box marks the average value and the box encloses measurements within the standard deviation. The horizontal line inside the box marks the median value.

Several batches were realized, and their performance is depicted in the statistical graph shown in Figure 9. Except for the fill factor, which had to be optimized, all parameters of Cs10 ANI were better than the other batches Cs10 CB. In Table 6, the PCE, hysteresis index and relative PCE percentage of initial efficiency over 90 days are reported.

### 3.4. Ideality Factor and Dark J-V

**Figure 10:**
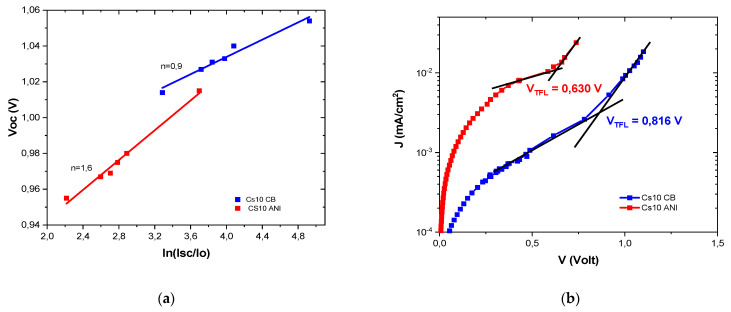
(**a**) Ideality factor Cs10 CB and Cs10 ANI; (**b**) dark J-V curves of the devices displaying the V_TFL_ point.

Information about the recombination can be obtained by estimating the ideality factor (n) of the solar cell, where an ideality factor close to 1 implies that all the recombinations are bimolecular and an ideality factor close to 2 implies that it is monomolecular and proceeding via trap-assisted recombination [25,26,27]. Under different light exposure, ions will migrate to different regions of solar cells and either make the conditions favorable to stabilize filled traps near the surface of the perovskite absorber layer or make the conditions favorable to induce a rapid depopulation of traps. When the traps are mostly filled, we expect a smaller fraction of trap-assisted recombination, longer charge diffusion lengths and an ideality factor closer to 1. When the traps are predominantly empty, we expect to have a higher fraction of trap-assisted recombination with a shorter diffusion length and a higher ideality factor. In the field of dye solar cells, non-integer ideality factors have been explained by the existence of a broad distribution of trap states combined with trap-limited recombination. For Cs10 ANI, the ideality factor indicates that both bimolecular and trap-assisted recombination are present, whereas for Cs10 CB, only bimolecular recombination is evident (Figure 10). The smaller ideality factor could be attributed to the band–band direct recombination, where a lower ion migration makes the Cs10 CB device more stable over time; indeed, after 90 days, the efficiency is approximately 90% of the initial value (see Table 5). At the same time, even the Cs10 ANI device, despite having a higher ideality factor, still maintained approximately 80% of the initial efficiency. This could be explained by calculating the trap density, N_t_, from the dark current–voltage curves, using the trap-filled limit voltage (V_TFL_) [21,28] and finding 1.99 × 10^16^ cm^−3^ and 2.57 × 10^16^ cm^−3^ for Cs10 ANI and Cs10 CB, respectively. This is the evidence of the defect passivation effect when ANI is used as an antisolvent, which improves the performance. Further investigation could be useful to obtain a clear vision of recombination dynamics, trap-assisted recombination and carrier diffusion lengths, as reported in the literature [29].

## 4. Conclusions

We explored the potential substitution of toxic CB with a safer alternative, anisole, in the fabrication of planar triple-cation perovskite solar cells using tin dioxide as an ETM. Anisole was effective in achieving functional PSCs comparable to those obtained with CB. Notably, the devices with anisole as an antisolvent exhibited even lower hysteresis, maintaining 80% of the initial PCE value over 90 days. The champion device, 10% CsI and anisole as antisolvent, showed a starting PCE of 20.2%. These findings reinforce the idea that environmentally friendly anisole can reliably replace toxic CB. We also varied the cesium concentration in the triple-cation precursor, which is known to enhance the stability. Perovskite devices, even though manufactured in a N_2_-filled glove box, were influenced by environmental conditions when they were electrically characterized in ambient air. A higher Cs concentration yielded more stable unencapsulated PSCs, which were less susceptible to fluctuating temperature and humidity. Moreover, we have stated that anisole can be used as an antisolvent because it is able to ensure a good growth of the perovskite film and is a sustainable alternative to chlorobenzene.

## Figures and Tables

**Table 1:** Properties of main antisolvents used for fabrication process of PSCs.

Antisolvent	Molecular Weight (g/mol)	Boiling Point (°C)	Viscosity (cP)	Solubility in H_2_O (g/100 g)	Relative Polarity	ICH Class
1-Butanol	74	118	3.006	7.7	0.586	3
Ethyl Acetate	88	77	0.443	8.7	0.228	3
Anisole	108	154	0.789	0.10	0.198	3
Chlorobenzene	113	131	0.760	0.05	0.188	2

**Table 2:** Papers where anisole was used as antisolvent for n-i-p architecture of triple cation perovskite devices.

Architecture (Glass/TCO)	J-V Parameters	Storage and Aging Time	Final Relative PCE	Ref.
compactTiO_2_/mesoTiO_2_/ Cs_0.05_(MA_0.17_FA_0.83_)_(0.95)_Pb(_I0.83_Br_0.17_)_3_	Voc = 1.15 V	<20% RH @ RT–1000 h	93%	Zhao et al. [8]
	Jsc = 21.98 mA/cm^2^			
	FF = 78%			
	PCE = 19.76%			
compactTiO_2_/meso TiO_2_/Cs_0.05_(MA_0.17_FA_0.83_)_(0.95)_Pb(_I0.83_Br_0.17_)_3_	Voc = 1.12 V	MPPT–60 s		Yavari et al. [9]
	Jsc = 23.26 mA/cm^2^			
	FF = 76%			
	PCE = 19.88%			
compactTiO_2_/meso TiO_2_/Cs_0.05_(MA_0.17_FA_0.83_)_(0.95)_Pb(_I0.83_Br_0.17_)_3_	Voc = 1.10 V	MPPT–45 s		Wang et al. [2]
	Jsc = 22.23 mA/cm^2^			
	FF = 75%			
	PCE = 18.43%			
compact TiO_2_/Cs_0.05_(MA_0.17_FA_0.83_)_(0.95)_Pb(_I0.83_Br_0.17_)_3_	Voc = 1.10 V	MPPT–100 s		Zhang et al. [11]
	Jsc = 22.78 mA/cm^2^			
	FF = 77%			
	PCE = 19.42%			
SnO_2_/FA_0.83_MA_0.117_Pb(I_0.87_Br_0.17_)_3et alet_	Voc = 1.14 V	MPPT–45 s		Habisreutinger et al. [10]
	Jsc = 22.07 mA/cm^2^			
	FF = 75%			
	PCE = 18.9%			
SnO_2_/FA_0.83_MA_0.117_Pb(I_0.87_Br_0.17_)_3_	Voc = 1.11 V	30% humidity @ 85 °C–500 h	50%	Pellaroque et al. [12]
	Jsc = 21.96 mA/cm^2^			
	FF = 73%			
	PCE = 18.2%			

**Table 3:** Bandgap and Urbach energies for all the samples at different cesium concentration and antisolvent.

Glass/ITO/SnO_2_/Perovskite	E_g_ (eV)	E_u_ (eV)
Cs10 CB	1.607	0.065
Cs 10 ANI	1.611	0.059
Cs5 CB	1.649	0.052
Cs5 ANI	1.647	0.051

**Table 4:** Temperature and relative humidity range from April to September.

Months	Temperature Range (°C)	Relative Humidity (%)
April	10–17	55–85
May	17–28	62–93
June	24–31	52–87
July	29–37	45–73
September	26–37	41–76

**Table 5:** J-V parameters (open-circuit voltage, V_oc_, short-circuit current density, J_sc_, fill factor, FF and power conversion efficiency, PCE, of champion devices of present work and * previous champion device [21], characterized in air environment with RH less than 30%).

Device	V_oc_ (mV)	J_sc_ (mA/cm^2^)	FF (%)	PCE (%)
Cs5 CB	1005	26.04	70.9	18.58
Cs5 CB *	1121	24.07	77.3	20.9
Cs5 ANI	1006	25.54	72.3	18.58
Cs10 CB	984	23.79	81.8	19.15
Cs10 ANI	1049	25.42	75.8	20.21

**Table 6:** Typical electrical performances, extrapolated from data of Figure 4, hysteresis index and PCE percentage of its initial efficiency over 90 days.

Glass/ITO/SnO_2_/Perovskite	PCE Reverse (%)	PCE Forward (%)	HI	Relative %PCE over 90 Days
Cs5 CB	17.03	13.85	0.18	62
Cs 5 ANI	17.10	16.10	0.06	80
Cs10 CB	18.28	16.64	0.09	92
Cs10 ANI	18.20	17.76	0.02	80

## Data Availability

Data are contained within the article.
